# Functional Connectivity of the Caudal Anterior Cingulate Cortex Is Decreased in Autism

**DOI:** 10.1371/journal.pone.0151879

**Published:** 2016-03-17

**Authors:** Yuanyue Zhou, Lijuan Shi, Xilong Cui, Suhong Wang, Xuerong Luo

**Affiliations:** 1 Mental Health Institute, the Second Xiangya Hospital, Central South University, Changsha, 410011, PR China; 2 Department of Child Psychiatry, Hangzhou Seventh People’s Hospital, Hangzhou, 310013, PR China; 3 Department of Neuroscience, the Third Affiliated Hospital of Soochow University, Changzhou, 213003, PR China; University Of Cambridge, UNITED KINGDOM

## Abstract

The anterior cingulate cortex (ACC) is frequently reported to have functionally distinct sub-regions that play key roles in different intrinsic networks. However, the contribution of the ACC, which is connected to several cortical areas and the limbic system, to autism is not clearly understood, although it may be involved in dysfunctions across several distinct but related functional domains. By comparing resting-state fMRI data from persons with autism and healthy controls, we sought to identify the abnormalities in the functional connectivity (FC) of ACC sub-regions in autism. The analyses found autism-related reductions in FC between the left caudal ACC and the right rolandic operculum, insula, postcentral gyrus, superior temporal gyrus, and the middle temporal gyrus. The FC (z-scores) between the left caudal ACC and the right insula was negatively correlated with the Stereotyped Behaviors and Restricted Interests scores of the autism group. These findings suggest that the caudal ACC is recruited selectively in the pathomechanism of autism.

## Introduction

The term Autism Spectrum Disorders (ASD) is a collective diagnosis characterized by substantial deficits in social communication, social interaction, and inflexible behavior, which was introduced in the Diagnostic and Statistic Manual of Mental Disorders (5^th^ ed.) (DSM-V)[1]. ASD encompasses the disorders of Autism, Asperger’s Syndrome (AS), Pervasive Developmental Disorder- Not Otherwise Specified (PDD-NOS), and other autistic conditions that were included in the DSM-IV. Individuals with ASD subtypes present heterogeneously, not only in terms of the severity of the core symptoms but also in the nature of symptoms, which range from primary sensory and motor functions to high-order social functions, including those related to the “theory of mind”. The anterior cingulate cortex (ACC) is an area that is anatomically and functionally related to frontal executive functions, parietal sensorimotor systems, and limbic intentional or emotional processes[2, 3]. The ACC can be divided into functionally distinct sub-regions. For example, Yu et al., divided the ACC into the subgenual ACC and the pregenual ACC, and demonstrated they were involved in an affective network, while the pregenual ACC also was correlated with the default-mode network[4]. Margulies et al., who mapped ACC functional connectivity during rest, demonstrated both a rostral/caudal distinction and a dorsal/ventral functional distinction [5]. It is thought that the ACC and its functionally distinct sub-regions may contribute to the neuropathology of ASD.

Accumulating evidence demonstrates that abnormalities in the structural or functional connections of the ACC and its sub-regions contribute to ASD. Barnea-Goraly et al. reported findings, based on diffusion tensor imaging, that fractional anisotropy (FA) values were reduced in the anterior cingulate, bilaterally, extending into the adjacent corpus callosum, the ventromedial frontal areas, and the subgenual prefrontal regions[6]. Thakkar et al. found that persons with ASD in their study showed significantly reduced FA in the rostral ACC and the dorsal ACC, bilaterally[7]. A study by Zikopoulos et al. of single axons in the white matter of the postmortem autistic brains, found changes below the ACC included a decrease in the largest axons that communicate over long distances, such as corticocortical connections, and an excessive number of thin axons that link neighboring areas, specifically, in the superficial white matter of the ACC[8].

Studies of the functional connections of the ACC have reported wide variations in group differences, probably because such differences depend on a number of factors, including, at least, whether the participants performed a task, what kind of task was employed, what mental processes were studied, and which ACC sub-regions were involved. When Kana et al. used a task requiring response inhibition, they found that the anterior cingulate gyrus, middle cingulate gyrus, and the insula of individuals with autism were functionally under-connected with the right inferior parietal lobe and the inferior frontal gyrus[9]. Conversely, Turner reported increased bilateral connectivity between the ACC and the caudate during visuomotor performance in adults with ASD[10]. Delmonte et al. also reported hyper-connectivity between the right ACC and the left caudate in response to social rewards in persons with ASD, which was associated with deactivation in the left caudate[11]. Analogously, Welchew et al. found abnormal functional integration of the anterior and dorsal cingulate cortex in AS, using permutation tests for inferences [12].

Investigators have started to study FC during rest in persons with ASD. Assaf et al., for example, found decreased FC between the precuneus and the anterior cingulate cortex, and the FC magnitude in these regions was inversely correlated with the severity of the patients' social mannerisms, as measured by the Autism Diagnostic Observation Schedule and the Social Responsiveness Scale [13].

Overall, the research literature suggests that the ACC has a prominent role in ASD and that altered FC between the ACC or its sub-regions and other brain areas may contribute substantially to this condition. However, since the ACC has functionally distinct sub-regions that have been defined inconsistently in previous studies, the results have varied, accordingly. Moreover, the relatively small sample sizes and using different inclusive diagnostic criteria in site-specific studies have been a challenge for exploring the neuropathology of autism. Thus, in the present study, we examined resting-state functional MRI data of 209 individuals with autism and 238 age-matched controls from the Autism Brain Imaging Data Exchange (ABIDE http://fcon_1000.projects.nitrc.org/indi/abide/) in an attempt to create a large clinically homogenous sample. In addition, we divided the ACC into five functionally distinct subdivisions, as done in a previous study, to calculate seed-based FC maps between the ACC and the rest of the brain[14].

Based on the existing literature, we hypothesized that the resting-state FC of the ACC would most likely be altered in the social and communication-related regions of the ACC (the rostral, perigenual, and subgenual ACC) in autism. Furthermore, to determine the potential index value of the FC of the ACC for the diagnosis and treatment of autism, correlations between the severity of autism symptoms, as measured by the Autism Diagnostic Observation Schedule (ADOS) and Autism Diagnostic Interview-Revised (ADI-R), and any FC alteration in the ACC were analyzed.

## Methods

### Participants

A total of 1,112 resting-state functional magnetic resonance imaging data sets were available from the Autism Brain Imaging Data Exchange (ABIDE). The data sets, which were scanned at multiple contributing sites, were comprised of 539 individuals with ASD and 573 age-matched typical controls. See the ABIDE websites and Di Martino et al[15]for details about assessment protocols, data provenance, phenotypic information, scanner acquisition parameters, and funding source.

### Imaging sample selection

The selection of the imaging sample in the current study was similar to that used in a previous study[16]. Briefly, the analysis in the present study was limited to: (1) males, because females were less than 10% of the total data set; (2) individuals who were clinically diagnosed with autism (whether confirmed with ADI-R or ADOS) and age-matched typical individuals; (3) sites in which full IQ (FIQ) was assessed for at least 75% of the subjects in each diagnostic group; (4) individuals with a FIQ falling within two standard deviations of the overall group mean; (5) individuals with a mean frame-wise displacement (FD) less than one standard deviation of the sample mean; (6) individuals with anatomical images having near-full brain coverage and successful registration; and (7) sites that had at least 10 subjects for each diagnostic group after meeting the above selection criteria. After applying these selection criteria, the data of 447 subjects (209 autistic and 238 typical individuals) from eight sites were used for resting-state imaging analysis. See more demographic information about these data sets in S1 and S2 Tables.

### Preprocessing

Image preprocessing was performed using statistical parametric mapping software (SPM8, Welcome Department of Imaging Neuro-Science, London, UK). The preprocessing steps included imaging correction for the acquisition delay between slices and head movements, normalization to the standard SPM8 echo-planar imaging template, and resampling to 3×3×3 mm^3^. Given that functional connectivity analysis is sensitive to small motions[17, 18], especially in persons with autism [19], spike regression was conducted on the normalized images to remove the effects of motion. Specifically, ‘bad’ volumes with a frame-wise displacement (FD) larger than 1 mm were included in the regression model[20]. Regression analysis also was conducted to remove the effects of other covariates, including 24-motion parameters, white matter signals, and Cerebrospinal Fluid (CSF) signals. The resulting images were smoothed with 8 mm full-width at half-maximum Gaussian kernel. Finally, the smoothed images were linearly detrended, and filtered at the range of 0.01–0.08 Hz.

### Functional connectivity analysis

**Fig 1:**
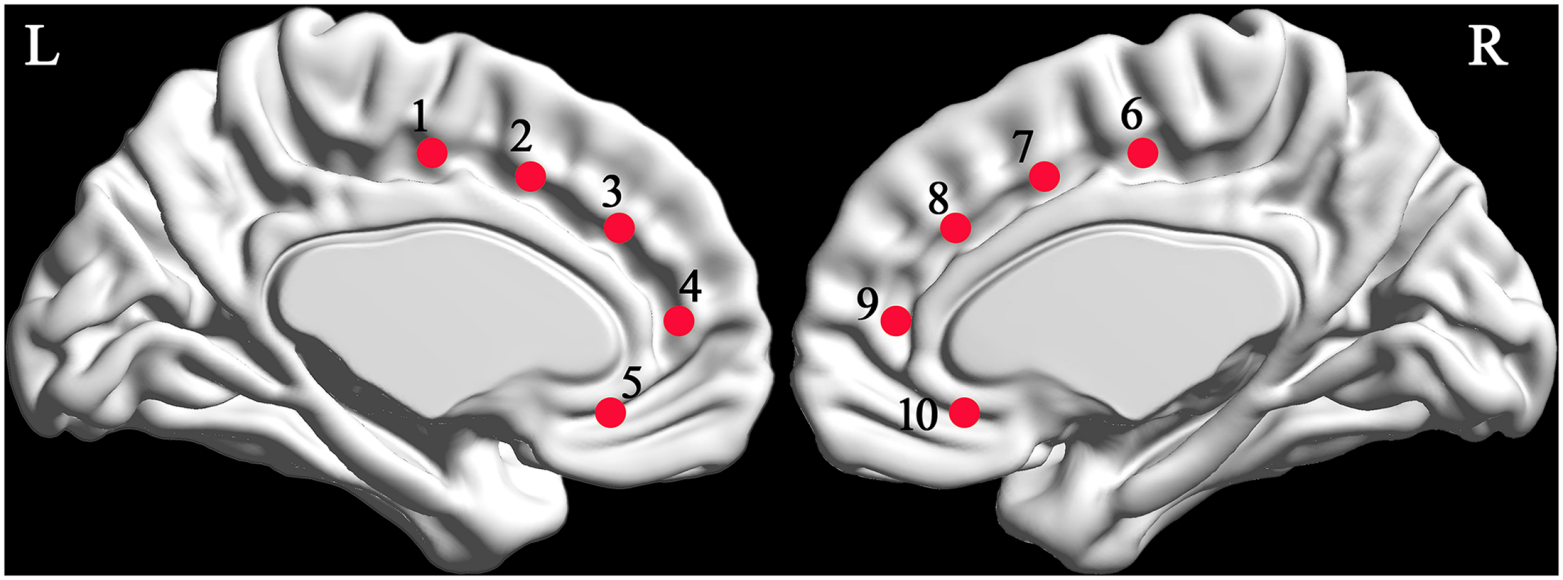
Locations of the five ACC sub-regions, which are labeled by red circles. Labels 1 to 10 indicate, respectively, the left caudal ACC, dorsal ACC, rostral ACC, perigenual ACC, and subgenual ACC, and the right caudal ACC, dorsal ACC, rostral ACC, perigenual ACC and subgenual ACC respectively. L, left; R, right; ACC, anterior cingulate cortex.

As suggested in previous studies[14], five bilateral MNI coordinates of the anterior cingulate cortex (ACC) were obtained and applied in the current study (Fig 1, Table 1). Then, we generated 10 spherical regions of interest (ROIs) with 3.5mm radii, based on these coordinates. The mean time-courses across voxels within each ROI were computed, which were then partially correlated with the time-courses of all the other voxels in the whole brain, while controlling for the effect of the other nine ROIs. The partial correlations were used to map the specificity of the connectivity of each ACC sub-region with cortical areas. This procedure generated the FC map for each group. These maps were normalized using the Fisher r-to-z transformation, and used in a second level analysis. For each ROI-based FC map of each group, we used a one-sample t-test with covariates, including site, FIQ, age, and FD, to test the hypothesis that the z-scores of each voxel were greater than zero. Multiple comparisons were corrected using Gaussian random theory, with q< 0.05 (voxel p < 0.01, z > 2.3). Only positive z-scores could survive, based on this threshold. Brain regions surviving the correction were combined into a mask across groups for each map. These masks were used in the subsequent two-sample t-tests.

**Table 1:** MNI coordinates of the five ACC sub-regions.

Seeds	ACC sub-regions	MNI coordinates		
		x	y	Z
Seed1	Caudal ACC	±5	-10	47
Seed 2	Dorsal ACC	±5	14	42
Seed 3	Rostral ACC	±5	34	28
Seed4	Perigenual ACC	±5	47	11
Seed 5	Subgenual ACC	±5	25	-10

ACC, anterior cingulate cortex.

In order to demonstrate that brain areas connected to ACC sub-regions are all specific from one another, we thresholded the T map with |T| > 15. The threshold could improve the representativeness of connectivity patterns of ACC sub-regions[21].

For each ROI-based functional connectivity map, two-tailed, two-sample t-test was implemented to characterize the difference in z-scores between the two groups, with covariance including age, site, FIQ, and FD. Multiple comparisons were corrected using the same method mentioned above.

Further, brain regions showing significant differences in z-scores between the two groups were evaluated using regions of interest (ROI) analysis. Those ROIs were generated by selecting spherical area with center at the peak of brain regions, and with 6 mm radius. A Pearson linear correlation was then conducted between the averaged z-score of those ROIs and the clinical variables listed in S1 Table for the autism group. Given that there were missing data for each clinical variable, subjects without the clinic data were not included in the correlation analysis. Notably, before correlation analysis, the effect of sites has been regressed out. The statistical level of p < 0.05 was regarded as significant.

## Results

**Fig 2:**
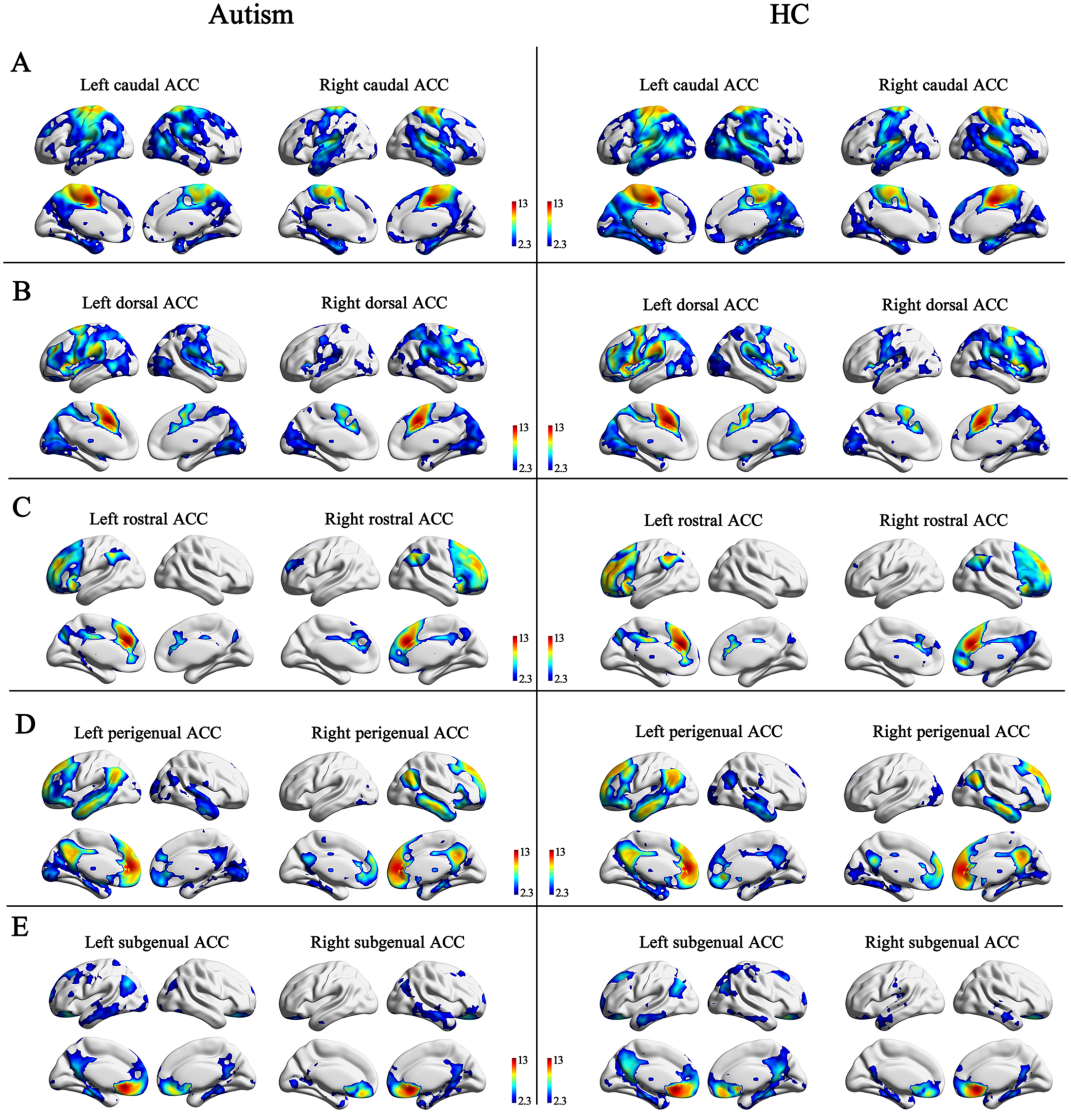
Functional connectivity maps of five ACC sub-regions for the ASD group and the healthy control group, corrected for multiple comparisons. The maps were corrected for multiple comparisons using Gaussian random theory with q < 0.05 (voxel p < 0.01, z > 2.3). The left panel (labels A to E) shows the functional connectivity maps of the caudal ACC, dorsal ACC, rostral ACC, perigenual ACC, and subgenual ACC in the autism group. The right panel (labels A to E) shows the functional connectivity maps of the right five ACC sub-regions in HC group. HC, healthy control; ACC, anterior cingulate cortex.

**Fig 3:**
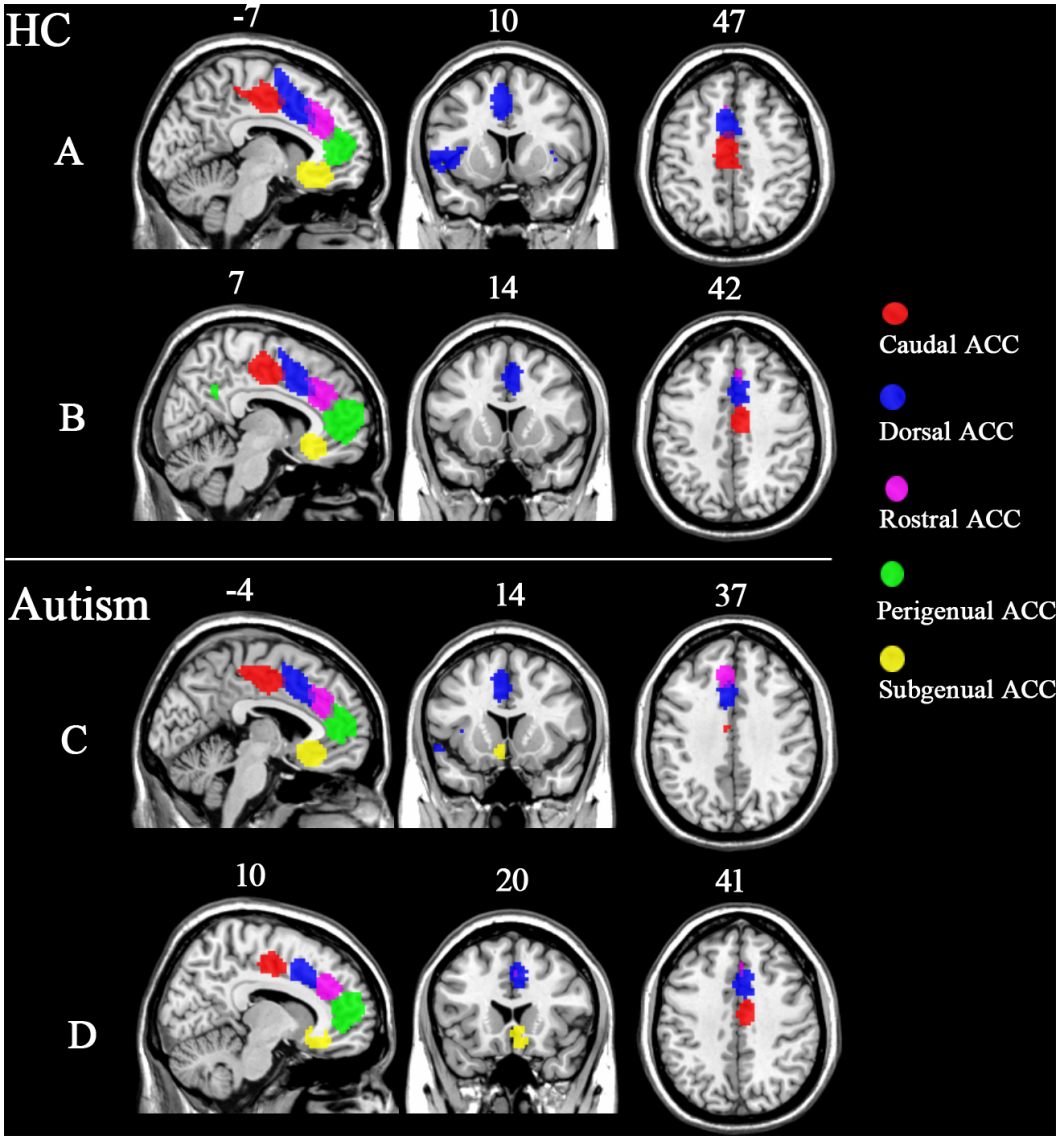
Functional connectivity maps of the five ACC sub-regions for the ASD group and the healthy control group, based on thresholding. Maps of the left five ACC sub-regions and the right five ACC sub-regions in the HC group (A, B) and the autism group (C, D), using a threshold of |t| > 15. The brain areas with different colors indicate the mask where the voxels survived the threshold.

One-sample t-tests revealed that the connectivity pattern of the ACC sub-regions was similar between the left and right hemispheres, and between the autism and HC groups. The caudal ACC was mainly connected with the somato-motor cortex, the visual cortex, and the temporal cortex. The dorsal ACC was mainly connected with the frontal cortex and the visual cortex. The rostral ACC was mainly connected with the frontal cortex and the precuneus. The perigenual ACC was mainly connected with the medial prefrontal cortex, post cingulate cortex, and temporal areas. The subgenual ACC was mainly connected with orbital frontal cortex and the post cingulate cortex (Fig 2). To ensure that subregions of the ACC had distinct projections to the cortex, we applied a threshold of |T| > 15. The connectivity pattern, therefore, differed slightly between the ACC sub-regions. Each of the ACC sub-regions was connected to brain areas close to the sub-region. In addition, the perigenual ACC was connected to the post cingulate cortex, and the dorsal ACC was connected to the insula (Fig 3).

**Fig 4:**
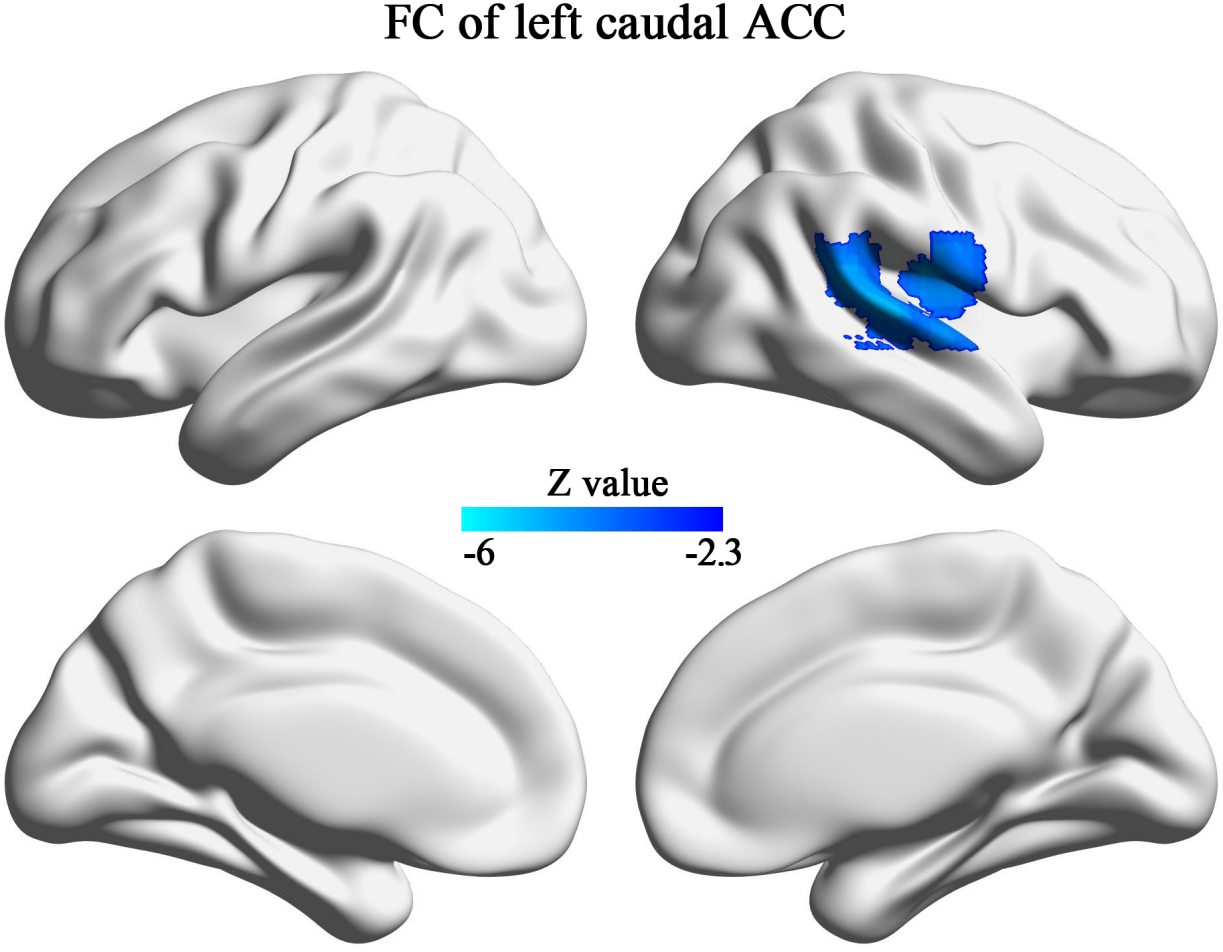
Brain regions showing significant differences in the functional connectivity of the left caudal ACC in the autism group compared with the healthy control group. Brain regions showing significant differences in functional connectivity of the left caudal ACC in persons with autism compared with the healthy controls. The blue color indicates decreased functional connectivity in the autism group. FC, functional connectivity; ACC, anterior cingulate cortex.

A significant group difference in the z-scores of the autistic and HC groups was observed between the left caudal ACC and the right rolandic operculum, right insula, postcentral gyrus, superior temporal gyrus, and the middle temporal gyrus (Fig 4, Table 2). We observed no difference in the z-scores of the right caudal ACC, the bilateral dorsal ACC, rostral ACC, perigenual ACC, or subgenual ACC.

**Table 2:** Functional connectivity alterations of the left caudal ACC in the autism group (n = 209) compared with the healthy control group (n = 238).

Brain regions (Autism < HC)	Voxel size	MNI coordinates			t-value at peak
		x	y	z	
The right rolandic operculum	69	45	-15	24	-5.8
The right insula	25	36	-15	15	-3.3
The right postcentral gyrus	20	57	-12	21	-3.2
The right superior temporal gyrus	174	66	-30	9	-4.4
The right middle temporal gyrus	27	63	-39	12	-3.1

The results were corrected for multiple comparisons using Gaussian random theory with q < 0.05 (voxel p < 0.01, z > 2.3). ACC, anterior cingulate cortex; HC, healthy controls

**Fig 5:**
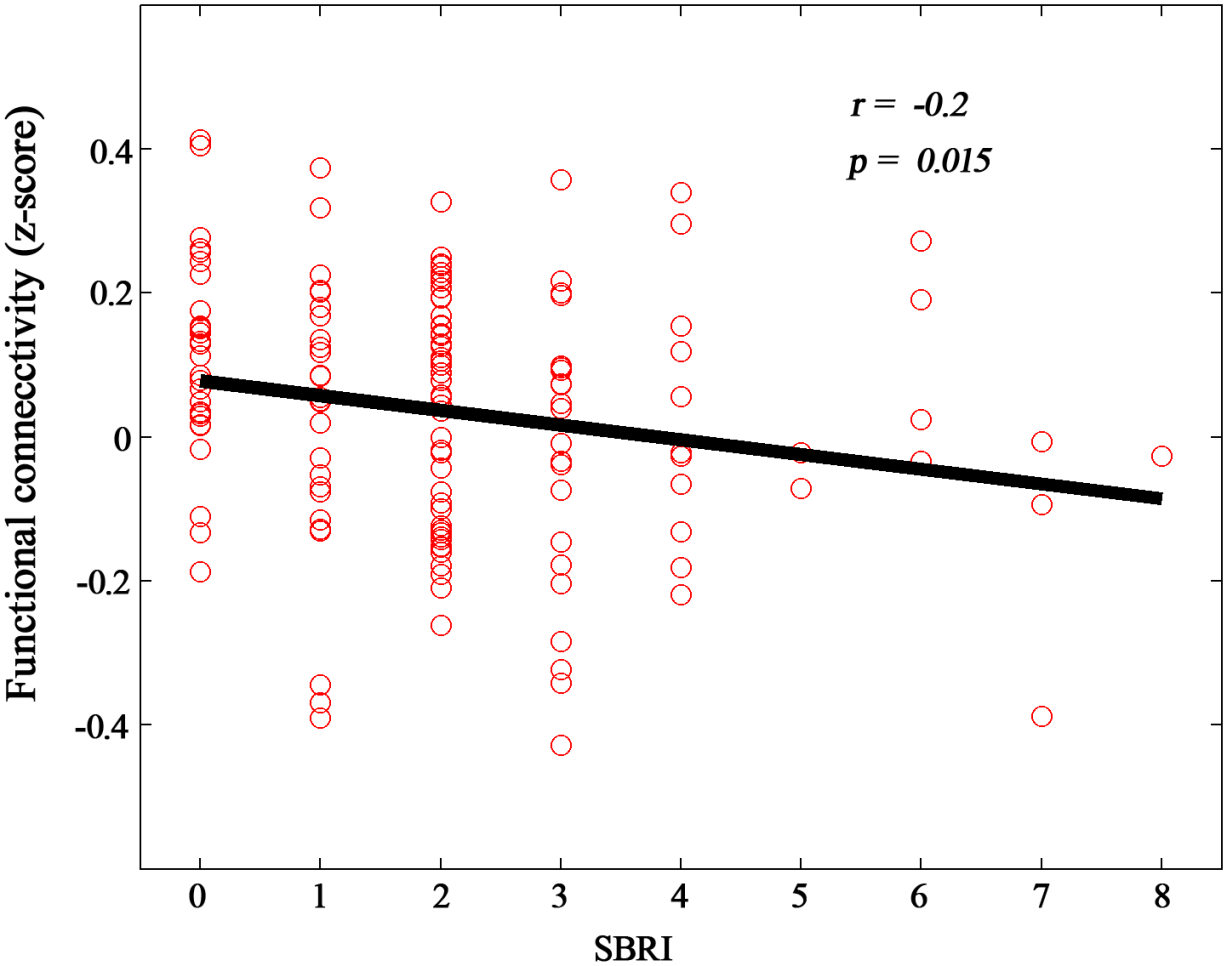
Correlation between altered functional connectivity and Stereotyped Behaviors and Restricted Interests (SBRI) scores in the autism group. Altered functional connectivity (z-scores) between the left caudal ACC and right insula were significantly correlated with SBRI scores in the autism group. ACC, anterior cingulate cortex.

We found a significant negative correlation (p < 0.05) between the FC (z-scores) of the left caudal ACC and the right insula and the Stereotyped Behaviors and Restricted Interests scores in the autism group (Fig 5, Table 3).

**Table 3:** Pearson correlations between functional connectivity (z-scores) and clinic variables in the autism group.

Clinic variables	Subject (N)	ROI (R value / P-value)				
		Right ROL	Right INS	Right PoCG	Right STG	Right MTG
ADI-R Scaled Scores						
Social	131	0.028/0.759	-0.051/0.565	-0.031/0.724	0.023/0.789	-0.029/0.746
Communication	132	0.101/0.252	0.0270.756	-0.021/0.811	0.009/0.920	0.078/0.373
RPB	132	0.018/0.841	0.033/0.706	-0.031/0.726	0.009/0.917	0.079/0.368
Onset	104	0.158/0.109	0.142/0.151	0.094/0.343	0.142/0.151	0.138/0.163
ADOS scaled Scores[22]						
Social & communication	154	0.114/0.158	0.066/0.418	-0.058/0.473	0.024/0.769	-0.021/0.800
communication	154	0.096/0.236	0.040/0.626	-0.048/0.472	-0.012/0.877	-0.011/0.896
social	154	0.109/0.176	0.072/0.375	-0.050/0.535	0.041/0.610	-0.023/0.773
SBRI	149	-0.002/0.980	**-0.200/0.015**	-0.078/0.353	-0.093/0.243	-0.157/0.057
ADOS scaled Scores[23]						
Social affect	104	0.052/0.603	0.042/0.675	-0.062/0.530	0.008/0.934	-0.017/0.861
RRB	104	0.110/0.265	-0.108/0.273	-0.062/0.532	-0.071/0.472	-0.056/0.571
Social affect & RRB	107	0.093/0.341	0.006/0.953	-0.046/0.636	-0.008/0.929	-0.028/0.776
Calibrated Severity	107	0.122/0.210	-0.007/0.938	-0.058/0.551	0.012/0.906	-0.035/0.718

The values in bold indicate significant correlations with p < 0.05. ROI, region of interest; R, correlation coefficient; P, p-value; RRB, restricted repetitive behaviors; SBRI, stereotyped behaviors and restricted interests; ROL, rolandic operculum; INS, insula; PoCG, postcentral gyrus; STG, superior temporal gyrus; MTG, middle temporal gyrus.

## Discussion

This study examined the resting-state functional connectivity of five equidistant axially distributed seeds in the ACC to analyze differences between individuals with autism and healthy controls. The results for both groups were partially consistent with previous studies. Our results demonstrated that the five sub-regions of the ACC are involved in different functional networks that are mainly distributed close to the sub-regions[5, 14, 24, 25]. Roughly similar connective patterns were found between the two groups, and there was no significant between-group difference in FC between ACC sub-regions and areas proximal to the ROIs. Several previous studies have suggested that ASD has a local over-connectivity pattern [10, 26]. For example, Di Martino et al. demonstrated increased functional connectivity between nearly all striatal sub-regions and heteromodal associative areas and the limbic cortex in ASD[27]. This result is consistent with findings that there were no between-group differences in within-network connectivity in relatively young boys with ASD[28], as well as with the results of largely typical resting-state functional connectivity in adults with ASD[19]. Thus, the local over-connected model of ASD was challenged.

### Decreased FC of the caudal ACC

Using samples selected from ABIDE, Di Martino et al. demonstrated both hypo- and hyper-connectivity in the intrinsic functional brain architecture of individuals with ASD compared to typical controls, and that hypo-connectivity was dominant for cortico-cortical and interhemispheric connectivity[15]. Vladimir et al. found lower functional connectivity between the anterior and posterior medial cortex in autism[29]. Similarly, our results found FC between the left caudal ACC and the right rolandic operculum, insula, postcentral gyrus, superior temporal gyrus, and the middle temporal gyrus, which are related mostly to the sensorimotor networks, was decreased in the autistic brain. It is increasingly accepted that brain areas that are functionally connected in the resting-state form intrinsic networks that are synergistically activated in performing the network’s typical function. Presumably, the decreased FC of sensorimotor-related networks in autism would produce deficits in their relevant functions. This is, indeed, true in clinical findings. Over 96% of persons with autism report hyper- and hypo-sensitivities in visual, auditory, tactile, and vestibular domains, as well as proprioception[30–33]. Alterations in sensory perception and primary motor function are not only ubiquitous features of autism, themselves, but they may underlie its core behavioral symptoms. Many children with autism are unresponsive to names but agitated by ordinary noises [34–36]. Deficits in the visual domain are particularly evident in autism. For example, limited eye-contact in early infants is a good predictor of a later diagnosis of autism[37, 38]. Toddlers with ASD also show an altered visual preference for nonsocial stimuli (e.g., dynamic geometric figures) rather than social stimuli (e.g., playing children) compared to the typical developing control[39, 40]. Individuals with autism engage in repetitive movements that are thought to produce sensory self-stimulation[41]. Cascio et al found that the ASD adults was under-responsive to the tactile stimuli (pleasant, neutral, and unpleasant) compared with the TD group while they had greater activation in the posterior cingulate and insula in response to the unpleasant tactile stimulus[42]. Our results showed significant between-group differences in the FC of the sensory-motor related caudal portion of the ACC rather than in the FC of the “social and emotional” related anterior portion of the ACC. Disruptions of connections of the caudal ACC with somatosensory and insula cortex could plausibly underlie altered awareness of emotions and feelings of self and others in autism. Thus further research on the specific components and interactions among the components of the sensorimotor networks that are impaired in autism could provide a comprehensive understanding about these potentially fundamental deficits in autism. Moreover, they may be used as valuable signs for diagnosing autism and for monitoring interventions for autism.

### FC between the caudal ACC and the insula associated with SBRI scores

The autistic stereotyped behavior and restricted interest include a broad range of behaviors from simple motor mannerisms to cognitive-involved behaviors including rigid insistence on routine, and being preoccupied by some kind of interests[22]. All SBRIs share the characteristic of either identical-pursuing or variation-avoiding and could be self-reinforced. These patterns of behavior could be related to a connection between sensory arousal and the subjective experience of pleasure. The insula is located at the transition of information about bodily arousal and the physiological state of the body to subjective feelings[43]. Research suggests the insula, with the anterior and posterior mid-cingulate cortices, is involved in interoceptive information processing of subjective representations of the body and skeletal-motor body orientation[44]. A number of functional imaging studies have shown that the insula is activated during the administration of abusive drugs and being exposed to drug cues, and that this activity is correlated with subjective urges[45, 46]. When someone compares seeing interesting objects that s/he owns with those owned by others, increased Blood Oxygen Level Dependent (BOLD) responses in the insula and the ACC distinguish between ASD and TC groups, and insula responses are associated with parental reports of the degree to which a child’s restricted interests interfere with the child’s daily life[47]. Some researchers have reported that adults with ASD show greater responses to unpleasant tactile stimuli in the post cingulate cortex and the insula, which were correlated with the severity of ASD symptoms[42]. Taken together, these results suggest that insula and ACC responses to emotionally related stimuli (whether positive or negative) are greater than those to control stimuli. The abnormal activation of the insula may have been due to an abnormal top—down process governed by the ACC. Turning to the present study, our results showed that functional connectivity between the left caudal ACC and the right insula in the autistic group was inversely associated with the severity of the autistic participants’ SBRS scores. This result cannot be directly related to previous research results. However, we do not think that they are conflicting. Like the ACC, the insula has been demonstrated to have functionally distinct areas with at least a posterior region associated with sensorimotor processes and anterior areas associated with empathy and cognitive functions. In addition, these sub-regions of the ACC and insula are anatomically and functionally connected to multiple functional networks, allowing these regions to flexibly participate in a wide range of functional processes. As Allman et al. suggested, in autism, fast intuitions may fail to be melded with slower, deliberative judgments because of abnormally developed Von Economo neurons, which relay the output of the fronto-insular and ACC to the parts of frontal and temporal cortex[48]. Moreover, it is not intended that the present result underlie the general ASD pathology. Further investigation is required to understand the finding that connectivity between the left caudal ACC and the right insula inversely correlated with restricted and repetitive behaviors. And further research should focused definitely on sub-regions of such brain areas and functional connectivity in the resting state should be interpreted in relation to task-state study results.

## Limitations

Several limitations of the present study need to be addressed. First, this study only included male subjects. Thus, the results may not be generalizable to females. Second, the FIQ of the samples in this study were around or above the average; thus, the results of this analysis may apply only to high-functioning persons with autism. Third, although the average age of the two groups were not different, there probably were individuals within each cohort who were in different developmental periods that differ significantly in terms of brain maturational processes, including differences in brain connectivity. Hence, functional connections in the social and emotional networks of the ACC sub-regions, which have relatively extended developmental courses[30, 49–52], may differ significantly within each group, thereby obscuring real between-group differences. Future studies should use smaller age and IQ ranges to reduce the heterogeneity for group-wise comparisons.

## Conclusion

This is the first study to examine the resting-state functional connectivity of five ACC subdivisions in persons with autism compared to age-matched healthy controls, using such a large sample. The results found reduced functional connectivity between the caudal ACC and sensorimotor areas and the insula, and its association with the severity of autistic symptoms. The results suggest that the seed-based networks of persons with autism were less integrated in the resting-state compared to networks of the healthy controls and, thus, their typical functions were impaired. Nevertheless, no significant between-group difference was found in local connections of the ACC sub-regions, which may be due to the characteristics of this study or may imply that further research is needed to support the altered functional connectivity hypothesis of ASD.

## Supporting Information

S1 Table Continuous demographic information of autism and healthy controls. (DOCX)

S2 Table Categorical demographic information of autism and healthy controls. (DOCX)
