# Supplementary material for: Functional Connectivity of the Caudal Anterior Cingulate Cortex Is Decreased in Autism
Source: PLoS One. 2016 Mar 17;11(3):e0151879. doi: 10.1371/journal.pone.0151879 (PMC4795711; doi:10.1371/journal.pone.0151879)
Supplement: S1 Table — (DOCX) [file pone.0151879.s001.docx]

**S1 Table.** Continuous demographic information of autism and healthy controls.

| Variables | Sites (N=8) | Ranges across sites  Autism (N=209) / HC (N=238) | | | | Grand Mean and SD  Autism (N=209) / HC (N=238) | | | Group  difference  (*p*-value) |
| --- | --- | --- | --- | --- | --- | --- | --- | --- | --- |
|  |  | Min | Max | Mean | SD | Sub n | Mean | SD |  |
| Age at scan (years) | 8 | 7.2-18/  6.5-18 | 14.9-50.2/ 13.6-39.4 | 12.8-23.2/ 12.1-23.4 | 1.1-7.8/ 1.2-7.4 | 209/238 | 17.1/16.7 | 7.1/6.2 | 0.5797 ^a^ |
| Full IQ | 7 | 78-89/  80-98 | 118-135/  126-139 | 96.3-109.7/  105.9-113.5 | 10.4-15.2/  8.9-12.8 | 198/222 | 103.7/111.1 | 13.5/11.5 | <0.0001 ^a^ |
| Verbal IQ | 8 | 69-88/  80-103 | 108-136/  125-147 | 95.9-109.8/  106.3-118.3 | 10.6-16.5/  9.2-13.2 | 209/238 | 102.6/112.5 | 14.7/12.3 | <0.0001 ^a^ |
| Performance IQ | 8 | 64-86/  67-97 | 122-149/  121-137 | 98.3-109/  102.1-111.9 | 11.5-19.3/  8.2-13.6 | 208/236 | 104.1/107.8 | 15.3/12.4 | 0.0052 ^a^ |
| Mean FD (mm) | 8 | 0.04-0.09/  0.04-0.12 | 0.26-0.78/  0.26-0.80 | 0.14-0.30/  0.12-0.24 | 0.05-0.20/  0.04-0.18 | 209/238 | 0.21/0.16 | 0.15/0.12 | <0.0001 ^a^ |
| ADI-R |  | | | | | | | | |
| Social | 5 | 7-12/  - | 27-28/  - | 20-21.8/  - | 3.8-5.6/  - | 131/  - | 20.4/  - | 4.8/  - | - |
| Communication | 5 | 4-10/  - | 22-25/  - | 15.5-16.4/  - | 3.1-5.6/  - | 131/  - | 16.1/  - | 4.2/  - | - |
| RRB | 5 | 0-4/  - | 10-12/  - | 5.7-7.4/  - | 2.2-2.7/  - | 131/  - | 6.5/  - | 2.5/  - | - |
| Onset | 4 | 0-2/  - | 5-5/  - | 3.0-3.6/  - | 1.0-1.5/  - | 104/  - | 3.4/  - | 1.3/  - | - |
| ADOS |  | | | | | | | | |
| Total Score  (social + communication) | 5 | 2-9/  - | 16-22/  - | 10.5-13.2/  - | 2.7-4.5/  - | 154  - | 12.3/  - | 3.8/  - | - |
| Social | 5 | 2-7/  - | 11-14/  - | 7.3-9.6/  - | 1.6-3.1/  - | 154/  - | 8.3/  - | 2.6/  - | - |
| Communication | 5 | 0-2/  - | 5-8/  - | 3.1-4.6/  - | 1.1-1.5/  - | 154/  - | 4.0/  - | 1.5/  - | - |
| SBRI | 5 | 0-1/  - | 6-8/  - | 1.3-2.6/  - | 1.4-1.7/  - | 149/  - | 2.0/  - | 1.7/  - | - |
| ADOS |  | | | | | | | | |
| Social Affect &  RRB Total | 5 | 2-10/  - | 18-27/  - | 10.6-16.5/  - | 2.9-5.7/  - | 107/  - | 12.6/  - | 5.1/  - | - |
| Social Affect | 5 | 2-7/  - | 13-20/  - | 7.8-13.3/  - | 2.2-4.5/  - | 104/  - | 9.6/  - | 4.1/  - | - |
| RRB | 5 | 0-1/  - | 6-8/  - | 2-3.5/  - | 1.4-2.0/  - | 104/  - | 2.8/  - | 1.8/  - | - |
| Calibrated Severity Score | 5 | 1-6/  - | 10-10/  - | 6.2-8.7/  - | 1.4-2.2/  - | 107/  - | 7.0/  - | 2.2/  - | - |

Ranges of minima, maxima, means and SD, as well as group mean and SD for continuous phenotypic variables for each group (autism and HC). Noting that the Min, Max, Mean, SD indicate the minimum, maximum, mean and standard deviation value of variables within each data site. SD, Standard deviation. HC, Healthy controls. FD, Frame-wise displacement. RRB, Restricted repetitive behaviors. SBRI, Stereotyped behaviors and restricted interests.

^a^ The p-value was calculated using two-tailed two-sample t-test

^b^ The p-value was calculated using chi-square test.
