# Supplementary material for: Functional Connectivity of the Caudal Anterior Cingulate Cortex Is Decreased in Autism
Source: PLoS One. 2016 Mar 17;11(3):e0151879. doi: 10.1371/journal.pone.0151879 (PMC4795711; doi:10.1371/journal.pone.0151879)
Supplement: S2 Table — (DOCX) [file pone.0151879.s002.docx]

**S2 Table.** Categorical demographic information of autism and healthy controls.

| Variables | Sites  (N) | Values (Autism/HC) | | | | | *p*-value  (group difference) |
| --- | --- | --- | --- | --- | --- | --- | --- |
|  |  | Min | Max | Sub n | n | % |  |
| Handedness (n right handed) | 8 | 8/10 | 43/72 | 209/238 | 171/215 | 85.1/92.7 | 0.0113 ^b^ |
| Eye Statues at R-fMRI  (n eyes open) | 8 | 0/0 | 48/64 | 209/238 | 170/192 | 81.3/80.7 | 0.8576 ^b^ |
| Medication Status  (n on meds) | 6 | 4/- | 13/- | 148/- | 53/- | 35.8/- | - |
| Comorbidity | 1 | 22/- | 22/- | - | - | - | - |

The minima, maxima, total and percentages for categorical variables for each group (autism and HC). Noting that the Min and Max indicate the minimum and maximum value of variables within each data site. The Sub n, n, and % indicate total number of subjects, number of subjects including the information and percentage of subjects, respectively. HC, Healthy controls.

^a^ The p-value was calculated using two-tailed two-sample t-test

^b^ The p-value was calculated using chi-square test.
